# Population genetics of *Liriomyza trifolii* (Diptera: Agromyzidae) and comparison with four *Liriomyza* species in China based on *COI*, *EF-1a* and microsatellites loci

**DOI:** 10.1038/s41598-019-53886-9

**Published:** 2019-11-28

**Authors:** Jing-Yun Chen, Ya-Wen Chang, Xiao-Tian Tang, Si-Zhu Zheng, Yu-Zhou Du

**Affiliations:** 1 School of Horticulture and Plant Protection & Institute of Applied Entomology, Yangzhou University, Yangzhou, 225009 China; 2 Suzhou Customs, Suzhou, 215000 China; 3 Joint International Research Laboratory of Agriculture and Agri-Product Safety, Yangzhou University, Yangzhou, 225009 China; 4 Present Address: Department of Entomology, Texas A&M University, College Station, TX 77843 USA

**Keywords:** Evolutionary genetics, Entomology, Agricultural genetics

## Abstract

*Liriomyza* is a large genus that includes polyphagous and invasive species (*L. trifolii, L. sativae*, and *L. huidobrensis*), and oligophagous species such as *L. Chinensis* in China. Effective control of these invasive and oligophagous species is not easy due to the fast invasion rate, interspecific competition, and pesticide resistance. In this study, we investigated population genetics of five *Liriomyza* species *L. trifolii, L. sativae, L. huidobrensis, L. bryoniae*, and *L. chinensis* based on *COI* and *EF-1a* genes, and microsatellite DNA. These five *Liriomyza* species revealed highly conservative characteristics in the *COI* gene among populations collected from different geographical regions and host plants. By contrast, the mutation rate of the *EF-1a* gene was higher than *COI*, and phylogenetic tree based on *EF-1a* showed that haplotypes of *L. trifolii* and *L. sativae* were not distinguished well. Genetic differentiation in microsatellite loci was obvious among the five species. Our results also indicated that geographic isolation had a greater impact on genetic differentiation in *L. trifolii* than the host plant. Populations of *L. trifolii* in China showed a high to moderate level of genetic differentiation and they had divided into two groups representing the coastal areas of southern China and northern regions. The genetic diversity of the southern group was higher than the northern group. We speculated that the invasion of *L. trifolii* likely occurred in southern regions of China and then spread northward. Bottleneck analyses revealed that the *L. trifolii* population in China was in a steady growth period.

## Introduction

*Liriomyza* is one of the largest genera belonging to the subfamily Phytomyzidae, family Agromyzidae, and order Diptera, consisting 330 described species^1–12^. Among these, 160 species are harmful in field crops or ornamental plants^13^, and 23 species are of great economic significance^8^.

Due to the small size, rapid interspecific competition, invasion rates, and adaptability, insects are sensitive to geographic isolation, hosts and phenological niches that cause species differentiation^14–16^. It has been speculated that host specialization has resulted in many new species in polyphagous *Liriomyza* that are highly adaptable to environmental stress^9,17,18^. To determine population genetic structure and migration patterns in *L. sativae*, several research groups analyzed different populations in China using fragments of ITS1 and β-tubulin genes and microsatellites^14,19,20^. In a study of Wang^21^, nuclear rDNA-ITS2 and mitochondrial *COI* sequences were used to analyze population differentiation in several invasive leafminer populations. It has been showed that populations of *L. trifolii* had separated into one clade representing the United States populations and a second clade for Asia-Europe populations, and a low level of differentiation was observed in domestic populations. However, mtDNA and ITS may not be the most suitable molecular markers for genetic differentiation analysis, because mitochondrial genes are highly conserved among intraspecific populations of insects and ITS is not suitable for analysis of intrageneric populations^14,15,20^. Microsatellites marker is a highly polymorphic co-dominant molecular marker with many characteristics, such as low requirements for DNA quality, good repeatability, simple detection methods, etc., and therefore it has been well applied in studies on population genetic structure, genetic relationship identification, genetic map construction and gene mapping to explore the population genetics, molecular systematics and ecology^15,22^. But there were a few researches using microsatellite marker technology to unfold the population genetic structure in *Liriomyza* especially for these invaded species^14^.

Previous studies on the population genetic structure of *Liriomyza* have generally involved only a single species^14^, with only a few comparative studies on genetic relationships among species^21^. In this study, we investigated intraspecies genetic differentiation in *L. trifolii* and interspecies variations among five species in *Liriomyza* in order to understanding better the species diversity during the geographic isolation and population expansion. Five species, namely *L. trifolii, L. sativae, L. huidobrensis, L. bryoniae*, and *L. chinensis*, were collected from 38 cities in 11 provinces of China. Population genetics differentiations of *Liriomyza* from different regions of China and host plants were evaluated using *COI*, *EF-1a* and microsatellite polymorphisms.

## Results

### Genetic differentiation of populations

Haplotype and nucleotide diversity of *COI* in *L. trifolii* populations was conserved with consistent characteristics among populations from different geographical regions and host plants. The maximum haplotype number among *L. trifolii* populations was three, and most haplotypes had only a single base difference. *L. sativae* populations showed slightly more diversity, and the maximum number of haplotypes among three *L. sativae* populations was six. The populations of *L. huidobrensis, L. bryoniae* and *L. chinensis* showed relatively low diversity (Table S1).

Haplotype and nucleotide diversity of *EF-1a* was relatively high among intraspecies (e.g. *L. trifolii*) as compared with *COI*. Ten haplotypes were found in the ZZJD, HLJD and HSFQ populations of *L. trifolii*, and the CSJD population had the highest nucleotide diversity. The other four *Liriomyza* spp. also showed relatively high diversity in *EF-1a* (Table S2).

The average observed number of alleles (Na) in *L. trifolii* populations ranged from 6.625 (DGJD) to 3.37 (HLJD). The average effective number of alleles (Ne) of *L. trifolii* populations ranged from 4.3885 (CXJD) to 1.9154 (SQNGMZ). The observed heterozygosity (Ho) values of ten *L. trifolii* populations were greater than 0.5, and the highest Ho was 0.6771 in the BLJD population; the remaining nine populations had Ho values less than 0.5 and the lowest was 0.3125 in the HLJD population. *L. huidobrensis*, *L. bryoniae* and *L. chinensis* had a low heterozygosity. Populations of different hosts in the same geographic region (DGQC and BLJD, NNQC and NNJD, SQJDMZ and SQNGMZ) showed a great degree of similarity in Na and Ho. Most populations were deviated from the Hardy-Weinberg equilibrium (Table S3).

### Phylogenetic analyses

**Figure 1:**
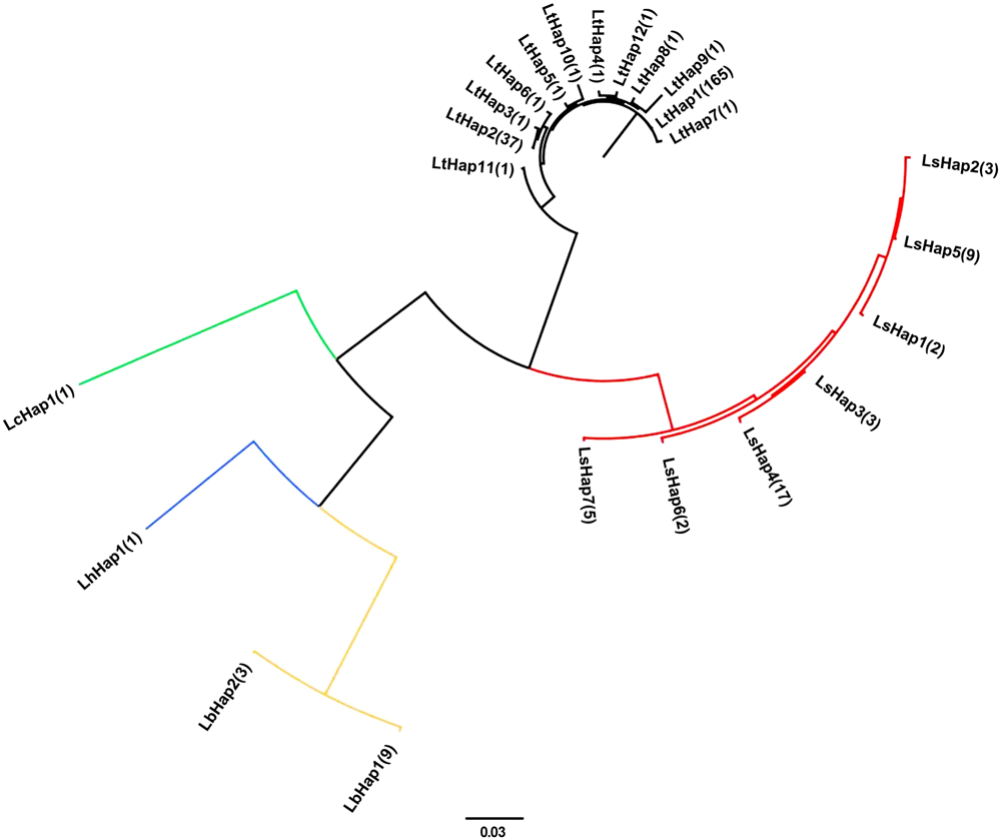
BI phylogenetic tree of five *Liriomyza* species based on *COI* haplotypes. Black lines represent *L. trifolii* haplotypes, red lines represent *L. sativae* haplotypes, green line represents *L. chinensis* haplotype, blue line represents *L. huidobrensis* haplotype, orange lines represent *L. bryoniae* haplotypes.

**Figure 2:**
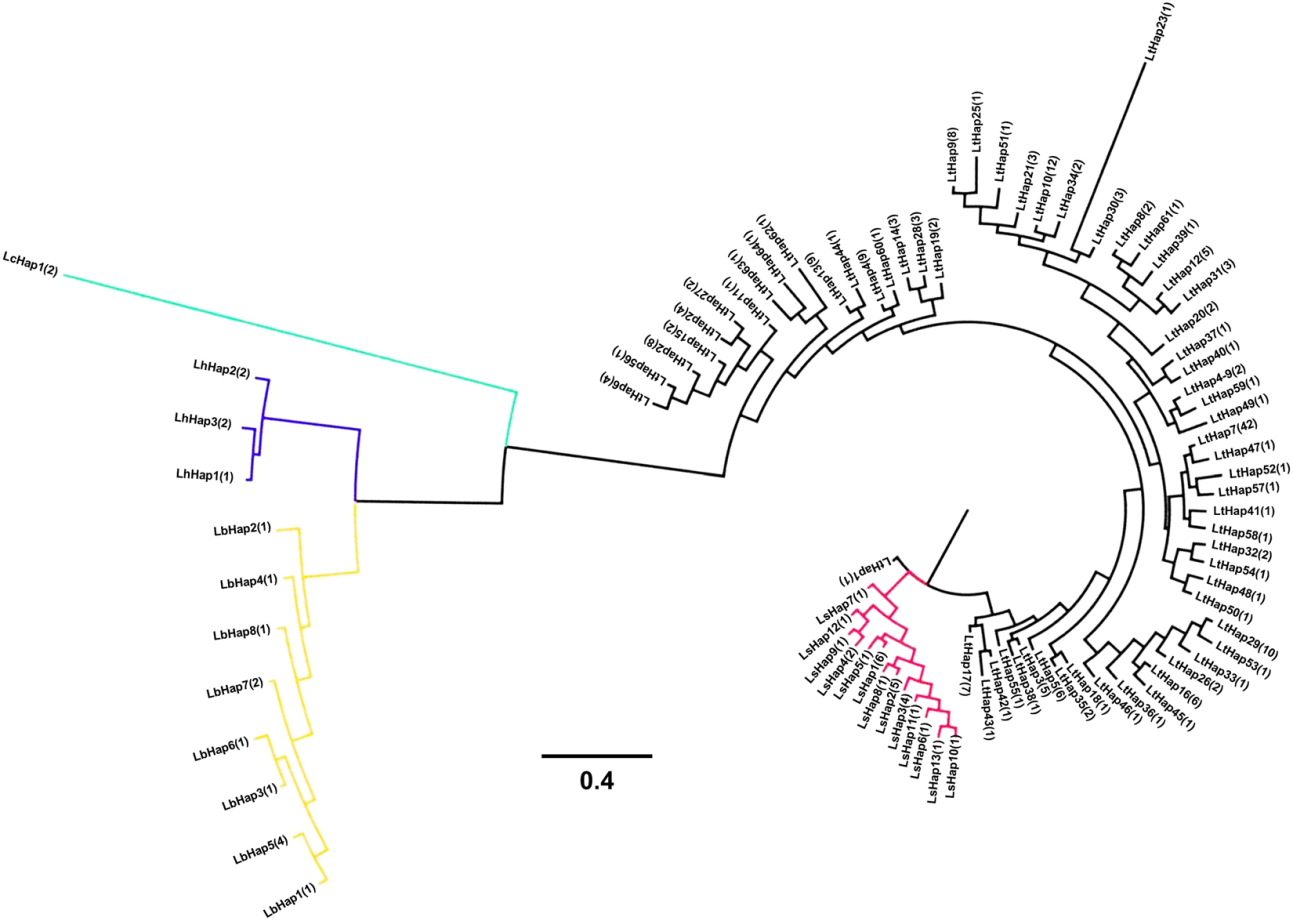
BI phylogenetic tree of five *Liriomyza* species based on *EF-1a* haplotypes. Black lines represent *L. trifolii* haplotypes, red lines represent *L. sativae* haplotypes, green line represents *L. chinensis* haplotype, blue lines represent *L. huidobrensis* haplotypes, orange lines represent *L. bryoniae* haplotypes.

The phylogenetic tree based on *COI* haplotypes (Fig. 1) showed that the five *Liriomyza* species had an obvious interspecific differentiation. The species relationship between *L. trifolii* and *L. sativae* were the most closest, and between *L. bryoniae* and *L. huidobrensis* was closer, while the relationships of *L. chinensis* with each of the other four *Liriomyza* species were distant. The phylogenetic tree based on *EF-1a* haplotypes (Fig. 2) was similar as the phylogenetic tree based on *COI* haplotypes, but haplotypes of *L. trifolii* and *L. sativae* were not distinguished well.

### Genetic differentiation–pairwise FST analyses

Because of the obvious interspecific differentiation of *COI* and *EF-1a* in the five *Liriomyza* species, only the intraspecific genetic differentiations based on *COI* and *EF-1a* genes in *L. trifolii* populations was analyzed. The results based on *COI* showed that the HLJD population exhibited high genetic differentiations from other 19 populations, and the highest differentiation was found between HLJD and BLJD (Table 1). The results based on *EF-1a* showed that the HBJD population exhibited high genetic differentiations from other 19 populations, and the HBJD population showed the highest differentiation with the CXJD (Table 2).

**Table 1:** Pairwise F_ST_ of *L. trifolii* populations based on *COI* gene.

population	DGQC	CZJD	SYJD	CSJD	CXJD	SQJD	HSJD	ZZJD	BLJD	WZJD	NNJD	NNQC	HLJD	HZQC	HDJD	JXQC	HNJC	HBJD
CZJD	**0.46351**																	
SYJD	—	**0.35906**																
CSJD	**0.49044**	—	**0.33692**															
CXJD	**0.34975**	—	0.24174	—														
SQJD	—	**0.47727**	—	**0.50829**	**0.36364**													
HSJD	—	**0.47727**	—	**0.50829**	**0.36364**	—												
ZZJD	0.16981	0.11515	0.10075	0.09504	—	0.18182	0.18182											
BLJD	—	**0.47727**	—	**0.50829**	**0.36364**	—	—	0.18182										
WZJD	—	**0.46351**	—	**0.49044**	**0.34975**	—	—	0.16981	—									
NNJD	0.08638	0.14318	—	0.11313	—	0.09774	0.09774	—	0.09774	0.08638								
NNQC	—	**0.31746**	—	**0.31542**	0.16952	—	—	—	—	—	—							
HLJD	**0.71734**	—	**0.57385**	—	0.13287	0.72727	0.72727	**0.34545**	**0.72727**	**0.71734**	**0.36152**	**0.57568**						
HZQC	—	0.23864	—	0.18852	0.13986	—	—	—	—	—	—	—	**0.38503**					
HDJD	—	**0.39644**	—	**0.40000**	**0.28205**	—	—	0.10954	—	—	—	—	**0.66798**	—				
JXQC	0.10811	—	0.07407	—	—	0.12003	0.12003	—	0.12003	0.10811	—	—	0.17585	—	—			
HNJC	—	0.17625	—	0.13479	0.06760	—	—	—	—	—	—	—	**0.34403**	—	—	—		
HBJD	—	**0.42424**	—	**0.42833**	**0.30303**	—	—	0.13636	—	—	0.06977	—	**0.65455**	—	—	0.10338	—	
HSFQ	0.08078	0.21739	—	0.20409	0.06250	0.09091	0.09091	—	0.09091	0.08078	—	—	**0.46591**	—	—	—	—	0.06061

— mean: Fst < 0.05, **bold** numbers mean: Fst **>** 0.25.

**Table 2:** Pairwise F_ST_ of *L. trifolii* populations based on *EF-1a* gene.

population	DGQC	CZJD	CSJD	CXJD	SQJD	HSJD	ZZJD	BLJD	WZJD	NNJD	NNQC	HLJD	HZQC	HDJD	JXQC	HNJC	HSFQ	HBJD
CZJD	0.10966																	
CSJD	0.11577	—																
CXJD	0.20945	0.12554	0.17216															
SQJD	0.21728	0.07304	0.13479	—														
HSJD	**0.25100**	0.11864	0.15803	—	—													
ZZJD	0.08690	—	—	0.17925	0.12388	0.18377												
BLJD	—	—	0.15116	**0.32650**	**0.33710**	**0.35377**	0.18828											
WZJD	0.05231	0.10142	0.10969	0.17853	0.16801	0.21793	0.07763	0.11499										
NNJD	—	—	0.0835	0.12624	0.12067	0.13991	0.10083	—	—									
NNQC	0.14211	0.10088	0.13483	—	—	—	0.12402	0.24757	0.06526	0.08119								
HLJD	0.15252	—	0.07976	—	—	—	—	**0.25837**	0.08475	0.10948	—							
HZQC	—	0.10978	0.13357	0.07806	0.11911	0.13827	0.14150	0.09289	0.08150	—	—	0.08751						
HDJD	0.22013	0.15526	0.12536	—	—	—	0.14075	**0.37403**	0.22749	0.14360	—	—	0.15585					
JXQC	0.05122	—	0.07210	—	—	—	—	0.15453	0.10316	—	—	—	—	—				
HNJC	—	0.05169	—	0.16013	0.14394	0.18007	—	0.10277	0.05635	—	0.10810	0.07978	0.08270	0.11934	—			
HSFQ	0.19163	0.06838	0.11359	—	—	—	0.09015	**0.28512**	0.14725	0.12118	—	—	0.13691	—	—	0.11804		
HBJD	**0.29091**	0.17021	0.12484	**0.43659**	**0.39726**	**0.40998**	0.13755	**0.37879**	**0.37394**	**0.30203**	**0.42984**	**0.27435**	**0.38793**	**0.39245**	0.18704	0.20289	**0.28556**	
SYJD	0.21152	**0.25191**	0.18070	**0.39878**	**0.35592**	**0.38515**	0.14841	**0.31311**	0.20383	0.17282	**0.34106**	**0.26812**	**0.35526**	**0.34494**	0.24762	0.14205	**0.26797**	**0.30171**

— mean: Fst < 0.05, **bold** numbers mean: Fst **>** 0.25.

In order to make clear interspecific and intraspecific nuclear genetic differentiations between five species of *Liriomyza*, pairwise F_ST_ scores of 25 populations (19 *L. trifolii*, three *L. sativae*, and one *L. huidobrensis*, *L. bryoniae* and *L. chinensis* populations) were compared based on 8 microsatellite loci (Table 3). Six pairwise F_ST_ values of *L. trifolii* populations were less than 0.05, and six were more than 0.25, and the other populations were between 0.05 and 0.25, indicating that most populations of *L. trifolii* were in a moderate level of genetic differentiation in China. The pairwise F_ST_ scores between the populations on different hosts in the same geographic region were 0.04457 for the NNQC and NNJD, 0.02928 for the DGQC and BLJD, 0.12234 for the HSFQ and HSJD, less than 0.05 for the SQJDMZ and SQNGMZ, and 0.08675 for the SQNGMZ and HNSGMZ, suggesting a lower genetic differentiation in microsatellite loci. The five *Liriomyza* species (especially *L. trifolii* vs. *L. sativae*) had high levels of interspecific genetic differentiation in microsatellite loci, although the species were similar in terms of morphology, niche occupation and feeding habits (Table 3).

### Population genetic structure

#### Analysis of population genetic structure based on eight microsatellite loci

**Figure 3:**
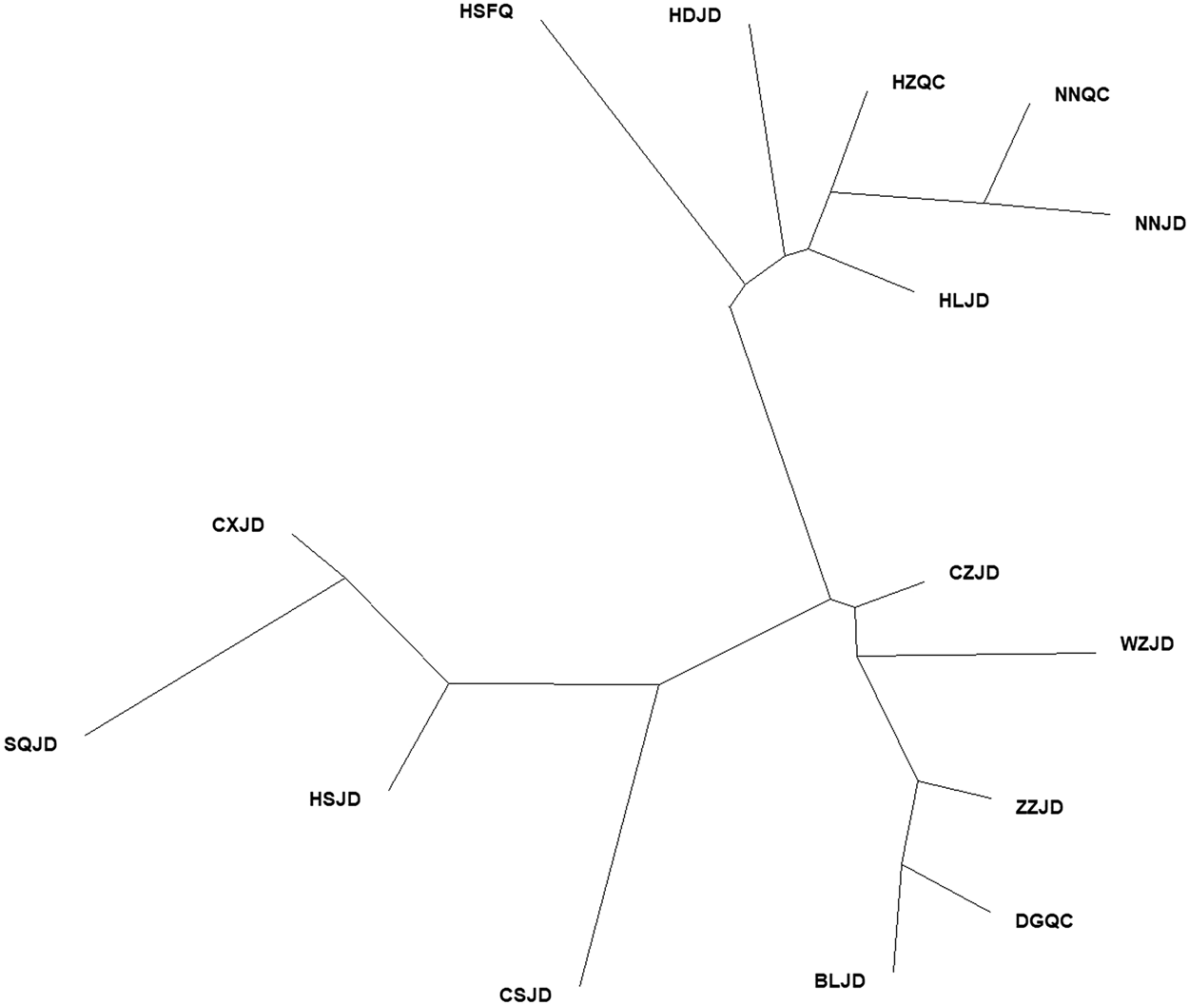
UPGMA dendrogram between *L. trifolii* populations based on Nei’s genetic distances. HSFQ: *Lycopersicon esculentum* population in Hengshui, HDJD: *Vigna unguiculata* population in Handan, HZQC: *Brassica chinensis* population in Huizhou, NNQC: *B. chinensis* population in Nanning, NNJD: *V. unguiculate* population in Nanning, HLJD: *V. unguiculate* population in Hangzhou, CZJD: *V. unguiculate* population in Changzhou, WZJD: *V. unguiculate* population in Wuzhou, ZZJD: *V. unguiculate* population in Zhangzhou, DGQC: *B. chinensis* population in Dongguan, BLJD: *V. unguiculate* population in Dongguan, CSJD: *V. unguiculate* population in Changshu, HSJD: *V. unguiculate* population in Hengshui, SQJD: *V. unguiculate* population in Shangqiu, CXJD: *V. unguiculate* population in Huzhou.

**Figure 4:**
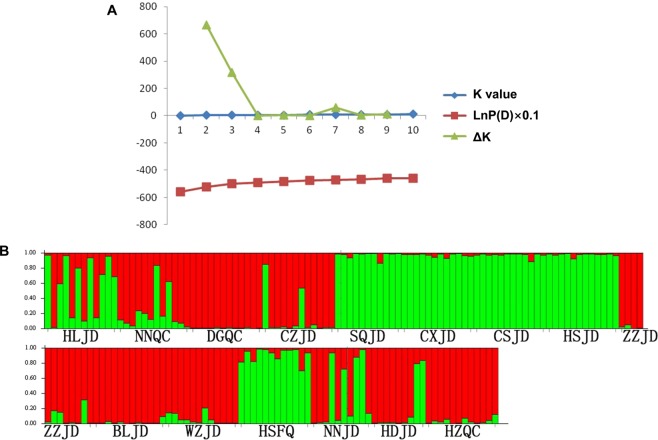
Clustering analysis by structure for full-loci dataset between *L. trifolii* populations. (**A**) Inference of the number of genetic cluster (K) for *L. trifolii* populations. (**B**) Proportion of the genome of each individual assigned to each of the two clusters. Each individual is represented by a vertical bar.

The phylogenetic tree of fifteen *L. trifolii* populations collected in two months in 2017 was constructed based on Nei’s genetic distances using UPGMA and the PHYLIP program. The UPGMA dendrogram (Fig. 3) showed that fifteen populations were basically clustered into two distinct main branches and four small scattered branches. Results of two population pairs NNQC/NNJD and DGQC/BLJD from different hosts in the same geographical region obviously converged to the nearest neighboring branch, which was consistent with pairwise F_ST_ analysis. However, the HSFQ/HSJD population pair did not converge. STRUCTURE analyses of the fifteen populations showed that the highest ΔK value was obtained for K = 2 (Fig. 4). Populations from coastal areas of southern China (DGQC, BLJD, ZZJD, HZQC) were assigned to one group (red portion of Fig. 4). Populations from Jiangsu and Zhejiang provinces and northern regions (CXJD, CSJD, SQJD, HSJD, HSFQ) were assigned to another group (green portion of Fig. 4).

#### Bottleneck test

Bottleneck analysis with populations of *L. trifolii* across China showed that none of these 19 *L. trifolii* populations exhibited heterozygosity under the stepwise mutation model (SMM), and there were only six populations (CXJD, CSJD, HSJD, HSFQ, ZZJD, WZJD) and nine populations with a statistically significant heterozygotes under the two-phase model (TPM) and the infinite allele model (IAM) (Table 3), respectively. These results indicated that the majority of *L. trifolii* populations did not undergo a genetic bottleneck and were in a steady growth period.

**Table 3:** Bottleneck test of *L. trifolii* populations based on eight microsatellite loci using IAM, TPM and SMM methods.

Population	IAM		TPM		SMM	
	Hde/Hex	Wilcoxon sign-rank test (H)	Hde/Hex	Wilcoxon sign-rank test (H)	Hde/Hex	Wilcoxon sign-rank test (H)
HLJD	2/5	0.23438	2/5	0.40625	2/5	0.46875
NNQC	1/7	0.09766	2/6	0.12500	4/4	0.72656
SYJD	1/7	**0.01953**	2/6	0.12500	2/6	0.23047
HBJD	3/5	0.37109	3/5	0.67969	4/4	0.72656
DGQC	5/3	0.57813	6/2	0.97266	6/2	0.99414
CZJD	4/4	0.19141	4/4	0.52734	6/2	0.96289
SQJD	1/7	**0.00977**	1/7	0.09766	4/4	0.32031
CXJD	0/8	**0.00195**	1/8	**0.00977**	3/5	0.09766
CSJD	0/8	**0.00195**	3/5	**0.03711**	4/4	0.52734
HSJD	1/7	**0.00391**	1/7	**0.00586**	1/7	0.09766
ZZJD	1/7	**0.00391**	2/6	**0.02734**	4/4	0.37109
BLJD	3/5	0.37109	7/1	0.98047	7/1	0.99414
WZJD	0/8	**0.00195**	2/6	**0.02734**	3/5	0.47266
HSFQ	1/7	**0.00586**	1/7	**0.00977**	2/6	0.12500
NNJD	1/7	**0.01953**	2/6	0.09766	2/6	0.23047
HDJD	2/6	0.27344	4/4	0.52734	4/4	0.67969
HZQC	3/5	0.37109	4/4	0.76953	6/2	0.97266
JXQC	2/5	0.18750	3/4	0.34375	3/4	0.65625
HNJC	3/5	0.23047	5/3	0.67969	5/3	0.84375

## Discussion

Population genetic structure and diversity are important factors affecting the survival and adaptability of invasive species. Population genetics in many pests were studied to find out their invasion and transmission routes^14,15,19–23^. In this study, the phylogenetic tree, pairwise F_ST_, and STRUCTURE analysis indicated that the degree of differentiation and direction of nuclear and mitochondrial genes were not completely consistent. *COI* in the five species of *Liriomyza* showed very conservative characteristics, but the mutation rate of *EF-1a* gene was relatively higher, and phylogenetic tree results showed that haplotypes of *L. trifolii* and *L. sativae* were not distinguished well. The results of microsatellite analysis showed that genetic distances among the five species of *Liriomyza* were significantly much longer than those within *L. trifolii* populations. In short, the five *Liriomyza* species showed high levels of genetic differentiation in mitochondrial and nuclear genes, and the interspecies differentiation in nuclear genes was obvious. *COI* and *EF-1a* gene were suitable molecular markers for interspecies genetic differentiation analysis and not for intraspecies of *Liriomyza* species, because *COI* as a mitochondrial gene and *EF-1a* as a reference gene are highly conserved among intraspecific populations of the five *Liriomyza* species. Microsatellites marker were suitable molecular markers for both interspecies and intraspecific genetic differentiation analysis of the five *Liriomyza* species, because microsatellite analysis showed both interspecies and intraspecific genetic differentiations among the five species of *Liriomyza*.

Spencer (1964) suggested that host specialization caused the development of many new species^5^. We found that geographic isolation had a greater influence on genetic differentiation within *L. trifolii*, which is consistent with previous results for *L. Sativae*^14^, but we did not find obvious influence of host plants on genetic differentiation in these species. We hypothesize that host plants have not yet driven reproductive isolation among populations, so the gene exchange among populations on different hosts occurs frequently.

The results of genetic differentiation and structure analysis showed that most populations of *L. trifolii* in China were in a high or moderate degree of genetic differentiation. Populations of *L. trifolii* could be divided into two groups, one from coastal areas of southern China and the other from northern China including Jiangsu and Zhejiang provinces. The genetic diversity of the southern group was higher than the other group, so the invasion of *L. trifolii* likely occured in southern regions of China and then spread toward northward. Bottleneck test analysis showed that the *L. trifolii* population in China was in a steady growth period, which was similar as *L. sativae*^14^. Genetic variation may lead to the rapid adaptation of insects to new environments and contributes to population establishment and spread. Our study has produced information on the geographical distribution of genetic variation of five *Liriomyza* species in China that may also help in management programs of these important pests.

## Materials and Methods

### Sample collection and DNA extraction

*Liriomyza* individuals (*n* = 281; Table 4) were collected and preserved in 100% ethanol at −20 °C until DNA extractions were performed. Genomic DNA was extracted from samples using the LabServ Tissue DNA Kit (Thermo Fisher Scientific, Massachusetts, USA) and then used for PCR.

**Table 4:** List of sample collection information.

Sample	Species	Collection location	Longitude	latitude	date	Host	Number
SYJD	*L. trifolii*	Sanya	109.51	18.25	2015–2017	*Vigna unguiculata*	12
HBJD	*L. trifolii*	Wuhan	114.34	30.55	2015–2017	*Vigna unguiculata*	12
DGQC	*L. trifolii*	Dongguan	113.74	23.01	2017.9.27	*Brassica chinensis*	12
CZJD	*L. trifolii*	Changzhou	119.90	31.63	2017.9.8	*Vigna unguiculata*	12
SQJD	*L. trifolii*	Shangqiu	115.70	34.51	2017.8.3	*Vigna unguiculata*	12
CXJD	*L. trifolii*	Huzhou	119.99	31.04	2017.9.8	*Vigna unguiculata*	12
CSJD	*L. trifolii*	Changshu	120.89	31.69	2017.9.6	*Vigna unguiculata*	12
HSJD	*L. trifolii*	Hengshui	115.51	38.00	2017.8.5	*Vigna unguiculata*	12
ZZJD	*L. trifolii*	Zhangzhou	117.69	24.55	2017.9.26	*Vigna unguiculata*	12
BLJD	*L. trifolii*	Dongguan	113.74	23.01	2017.9.27	*Vigna unguiculata*	12
WZJD	*L. trifolii*	Wuzhou	111.23	23.43	2017.9.28	*Vigna unguiculata*	12
NNQC	*L. trifolii*	Nanning	108.42	22.86	2017.9.29	*Brassica chinensis*	12
HLJD	*L. trifolii*	Hangzhou	120.02	30.39	2017.9.8	*Vigna unguiculata*	12
HSFQ	*L. trifolii*	Hengshui	115.51	38.00	2017.8.5	*Lycopersicon esculentum*	12
NNJD	*L. trifolii*	Nanning	108.42	22.86	2017.9.29	*Vigna unguiculata*	10
HDJD	*L. trifolii*	Handan	114.59	36.45	2017.8.6	*Vigna unguiculata*	9
HZQC	*L. trifolii*	Huizhou	114.40	22.93	2017.9.27	*Brassica chinensis*	12
JXQC	*L. trifolii*	Nanchang	115.91	28.67	2014–2017	*Apium graveolens*	12
HNJC	*L. trifolii*	Qionghai	110.47	19.26	2014–2017	*Brassica juncea*	12
HNSGMZ	*L. sativae*	Luoyang	112.57	34.76	2017.8.8	*Luffa cylindrica*	10
SQJDMZ	*L. sativae*	Shangqiu	115.70	34.51	2017.8.3	*Vigna unguiculata*	12
SQNGMZ	*L. sativae*	Shangqiu	115.70	34.51	2017.8.3	*Cucurbita moschata*	12
NMB	*L.huidobrensis*	Laboratory	103.28	25.52	2016	*Apium graveolens*	6
HNJDFQ	*L. bryoniae*	Xinxiang	113.80	35.10	2017.8.6	*Vigna unguiculata*	12
CB	*L. chinensis*	Shangqiu	115.70	34.51	2017.8.3	*Allium fistulosum*	6

### Primers and microsatellite markers

The primers for mtDNA *COI* gene were referred to Simon *et al*.^24^. Specific primers for *EF-1a* gene and eight microsatellite primers were designed in this study (Supplementary Table S4). A fluorophore (FAM, ROX, HEX or TAMRA) was included at the 5’ end of each pair of microsatellite primers (Supplementary Table S5) used for genotyping. All the primers used in this experiment were synthesized by GENEWIZ Inc (Suzhou, China), and microsatellite genotyping was performed by GENEWIZ Inc.

### PCR amplification and sequencing

The *COI* (n = 268; Supplementary Table S1) and *EF-1a* PCR (n = 252; Supplementary Table S2) of *Liriomyza* individuals (Table 4) were successfully amplified and sequenced. The amplification conditions were as follows: initial denaturation for 4 min at 94 °C, followed by 35 cycles of denaturation for 30 s at 94 °C, annealing for 30 s at 58 °C, elongation for 50 s at 72 °C, and a final extension step of 72 °C for 5 min. The microsatellite amplification of *Liriomyza* individuals (*n* = 281; Table 4) conditions were as follows: initial denaturation for 4 min at 94 °C, followed by 35 cycles of denaturation for 30 s at 94 °C, annealing for 30 s at 51–58 °C, elongation for 30 s at 72 °C, and a final extension step of 72 °C for 5 min. All amplified products were sequenced and genotyped by GENEWIZ Inc.

### Data analysis of *COI* and *EF-1a*

The *COI* and *EF-1a* sequences were preliminarily aligned using the CLUSTALW program^25^. Haplotype diversity (h), nucleotide diversity (p), and the mean number of pairwise differences were calculated to estimate DNA polymorphism using DnaSP 5.0^26^. Analysis of F-statistics (Fst) and genetic differentiation were performed using Arlequin v. 3.5^27^ with 10,000 permutations. Phylogenetic relationships were deduced by Bayesian inference (BI) and maximum likelihood (ML). Phylogenetic trees were constructed using MrBayes v. 3.1.1^28^ and a PHYML online web server^29^. For BI, nucleotide alignments were constructed using the MrBayes program with 20,000,000 generations and with the first 5000 discarded as burn-in. Support values for trees generated by BI were expressed as Bayesian posterior probabilities in percentages. ML analysis was performed by Mega 6.0^30^. Tree information was visualized and edited using Treeview. The haplotype network was performed using NETWORK v. 4.6^31,32^.

### Data analysis of microsatellites

Fundamental genetic parameters were calculated for all eight loci using POPGENE v. 3.2^33^ including the number of alleles (Na), the effective number of alleles (Ne), and observed (Ho) and expected heterozygosity (H_E_), as well as Nei’s genetic distance and genetic similarity. Deviation from Hardy-Weinberg equilibrium and linkage disequilibrium at each locus were calculated using GenePop v. 4.0 (http://wbiomed.curtin.edu.au/genepop/). The polymorphic information content (PIC) was calculated using Cervus 2.0^34^. Differentiation indices (F_ST_) were calculated using ARLEQUIN 3.5^27^. A phylogenetic tree based on Nei’s genetic distance was constructed using the unweighted pair group with the arithmetic mean (UPGMA) method of PHYLIP v. 3.69^35^. Bootstrap values were calculated using 1000 replicates. To assess the population genetic structure, we used Bayesian model-based clustering analysis with STRUCTURE v. 2.3.3^36^. We specified an initial range of potential genotype clusters (K) from 1 to 10 under the admixed model and the assumption of correlated allele frequencies among populations. For each value of K, ten runs were performed with 100,000 iterations discarded as burn-in followed by an additional 10,000 iterations. The most probable number of K values in the data was detected by comparing the log probability of the data lnP (D) for each value of K across all ten runs of Structure and by examining the standardized second-order change of lnP (D) and ΔK^37^. For selected K values, CLUMPP v 1.1.2^38^ was used to align cluster membership coefficients from ten replicates of cluster analyses using the Greedy algorithm with 10,000 random input orders; the results were then graphically displayed with DISTRUCT v. 1.1^39^. We also analyzed our data with the GENELAND package^40^ to further investigate the number of populations and the spatial location of genetic discontinuities between them. K was allowed to vary (1 to 10) with 100,000 MCMC iterations, and uncertainty was attached to spatial coordinates fixed to 1 km, and then the fixed modal K was obtained with the other parameters unchanged. A potentially significant heterozygosity excess (the signature of a bottleneck) was detected using a Wilcoxon signed rank test, as implemented in Bottleneck v. 1.2^41^. When a population experiences a reduction of its effective size, it generally develops a heterozygosity excess at selectively neutral loci. Previous analyses have shown that the most useful markers for bottleneck detection are those evolving under IAM, and they provide guidelines for selecting sample sizes of individuals and loci^41–44^; meanwhile, the TPM is thought to more closely simulate microsatellite mutation^45^. Unlike the SMM, which predicts all mutations corresponding to the increment or decrement of a single base-pair repeat, the TPM predicts the occurrence of an occasional multiple base-pair repeat^42^. The strict SMM is obviously the most conservative model for testing for a significant heterozygosity excess caused by bottlenecks, because in some conditions it can produce a heterozygosity deficiency, and due to the heterozygosity excess it is always lower than other mutation models. Because the actual mutation model followed by our microsatellites is unknown, we ran the program Bottle neck under the IAM, SMM, and TPM to determine whether these populations recently experienced a population decline or not.

## Supplementary information

Supplementary Tables
