# Supplementary material for: Population genetics of Liriomyza trifolii (Diptera: Agromyzidae) and comparison with four Liriomyza species in China based on COI, EF-1a and microsatellites loci
Source: Sci Rep. 2019 Nov 28;9:17856. doi: 10.1038/s41598-019-53886-9 (PMC6882889; doi:10.1038/s41598-019-53886-9)
Supplement: Supplementary file 1 — Supplementary Tables [file 41598_2019_53886_MOESM1_ESM.docx]

### Population genetics of *Liriomyza trifolii* [(Diptera: Agromyzidae)](http://www.baidu.com/link?url=NaxZ7Z1hv5E_fGQdEgv1Q9NvE41mQBhJ0Khr-v4PLGoxb-BPGm-p6Xu3qJuJVRZ1PRnPAftdmof-ulRQG4Nxsy1ifZq9NE7Ehh_TrIB50ashgQzV6U5PdmPtDv4vOh_Y) and comparison with four *Liriomyza* species in China based on *COI*, *EF-1a* and microsatellites loci

Jing-Yun Chen, Ya-Wen Chang, Xiao-Tian Tang, Si-Zhu Zheng, Yu-Zhou Du

**Supplementary Table S1.** COI haloptype diversity in all populations to be tested

| **Population** | **Sample Number** | **Haplotype** | **Haplotype diversity** | **pi** | **K** |
| --- | --- | --- | --- | --- | --- |
| SYJD | 10 | 3 | 0.378 | 0.00055 | 0.400 |
| HBJD | 12 | 2 | 0.167 | 0.00023 | 0.167 |
| DGQC | 11 | 1 | 0.000 | 0.00000 | 0.000 |
| CZJD | 12 | 3 | 0.621 | 0.00097 | 0.697 |
| SQJD | 12 | 1 | 0.000 | 0.00000 | 0.000 |
| CXJD | 12 | 2 | 0.530 | 0.00074 | 0.530 |
| CSJD | 7 | 3 | 0.714 | 0.00119 | 0.857 |
| HSJD | 12 | 1 | 0.000 | 0.00000 | 0.000 |
| ZZJD | 12 | 2 | 0.409 | 0.00057 | 0.409 |
| BLJD | 12 | 1 | 0.000 | 0.00000 | 0.000 |
| WZJD | 11 | 1 | 0.000 | 0.00000 | 0.000 |
| NNQC | 11 | 2 | 0.182 | 0.00025 | 0.182 |
| HLJD | 12 | 2 | 0.409 | 0.00057 | 0.409 |
| HSFQ | 12 | 2 | 0.303 | 0.00042 | 0.303 |
| NNJD | 11 | 3 | 0.473 | 0.00071 | 0.509 |
| HDJD | 7 | 1 | 0.000 | 0.00000 | 0.000 |
| HZQC | 12 | 3 | 0.318 | 0.00185 | 1.333 |
| JXQC | 10 | 3 | 0.600 | 0.00176 | 1.267 |
| HNJC | 12 | 3 | 0.318 | 0.00137 | 0.985 |
| HNSGMZ | 10 | 6 | 0.864 | 0.00361 | 2.606 |
| SQJDMZ | 12 | 6 | 0.758 | 0.00303 | 2.182 |
| SQNGMZ | 12 | 3 | 0.591 | 0.00254 | 1.833 |
| NMB | 6 | 1 | 0.000 | 0.00000 | 0.000 |
| HNJDFQ | 12 | 2 | 0.409 | 0.00057 | 0.409 |
| CB | 6 | 1 | 0.000 | 0.00000 | 0.000 |

**Supplementary Table S2.** EF-1a haloptype diversity in all populations to be tested

| **Population** | **Sample Number** | **Haplotype** | **Haplotype diversity** | **pi** | **K** |
| --- | --- | --- | --- | --- | --- |
| SYJD | 11 | 5 | 0.782 | 0.00305 | 2.182 |
| HBJD | 12 | 5 | 0.864 | 0.00248 | 1.773 |
| DGQC | 11 | 7 | 0.909 | 0.00413 | 2.955 |
| CZJD | 12 | 9 | 0.955 | 0.00358 | 2.561 |
| SQJD | 12 | 8 | 0.909 | 0.00265 | 1.894 |
| CXJD | 12 | 8 | 0.894 | 0.00284 | 2.030 |
| CSJD | 8 | 6 | 0.929 | **0.00983** | **7.036** |
| HSJD | 12 | 6 | 0.855 | 0.00345 | 2.473 |
| ZZJD | 12 | **10** | **0.970** | 0.00391 | 2.803 |
| BLJD | 12 | 6 | 0.864 | 0.00273 | 1.955 |
| WZJD | 11 | 9 | 0.909 | 0.00463 | 3.318 |
| NNQC | 12 | 7 | 0.879 | 0.00245 | 1.758 |
| HLJD | 12 | **10** | **0.970** | 0.00383 | 2.742 |
| HSFQ | 12 | **10** | **0.970** | 0.00459 | 3.288 |
| NNJD | 11 | 7 | 0.818 | 0.00325 | 2.327 |
| HDJD | 6 | 4 | 0.867 | 0.00289 | 2.067 |
| HZQC | 12 | 8 | 0.894 | 0.00375 | 2.682 |
| JXQC | 11 | 9 | 0.964 | 0.00498 | 3.564 |
| HNJC | 7 | 6 | 0.952 | 0.00665 | 4.762 |
| HNSGMZ | 9 | 5 | 0.861 | 0.00225 | 1.611 |
| SQJDMZ | 11 | 9 | 0.964 | 0.00295 | 2.109 |
| SQNGMZ | 4 | 3 | 0.833 | 0.00396 | 2.833 |
| NMB | 6 | 3 | 0.800 | 0.00168 | 1.200 |
| HNJDFQ | 12 | 8 | 0.894 | 0.00330 | 2.364 |
| CB | 2 | 1 | 0.000 | 0.00000 | 0.000 |

**Supplementary Table S3.** Genetic diversity of five *Liriomyza* species based on eight microsatellite loci

| **Locus** |  | **SSR-26** | **SSR-6** | **SSR-35** | **SSR39** | **SSR40** | **SSR-24** | **JY46** | **JY70bB** | **Average** |
| --- | --- | --- | --- | --- | --- | --- | --- | --- | --- | --- |
| **DGQC**  **n=12** | **Na** | **4.0000** | **2.0000** | **9.0000** | **7.0000** | **6.0000** | **6.0000** | **5.0000** | **9.0000** | **6.0000** |
|  | Ne | 2.3040 | 1.6000 | 4.4308 | 4.8000 | 3.1648 | 2.7961 | 2.1022 | 5.0526 | 3.2813 |
|  | Ho | 0.3333 | 0.0000 | 0.5000 | 0.7500 | 0.4167 | 1.0000 | 0.1667 | 0.8333 | 0.5000 |
|  | H_E_ | 0.5906 | 0.3913 | 0.8080 | 0.8261 | 0.7138 | 0.6703 | 0.5471 | 0.8370 | 0.6730 |
|  | P-HW | 0.0621 | **0.0016**** | **0.0066**** | **0.0018**** | **0.0037**** | **0.0019**** | **0.0003**** | 0.8583 | / |
| **BLJD**  **n=12** | Na | **5.0000** | **5.0000** | **7.0000** | **8.0000** | **8.0000** | **7.0000** | **5.0000** | **8.0000** | **6.6250** |
|  | Ne | 2.7692 | 2.2857 | 5.4340 | 4.9655 | 4.5714 | 4.2353 | 2.2500 | 4.2353 | 3.8433 |
|  | Ho | 0.5833 | 0.4167 | 0.6667 | 0.5833 | 1.0000 | 1.0000 | 0.5833 | 0.5833 | 0.6771 |
|  | H_E_ | 0.6667 | 0.5870 | 0.8514 | 0.8333 | 0.8152 | 0.7971 | 0.5797 | 0.7971 | 0.7409 |
|  | P-HW | 0.1809 | 0.0795 | **0.0030**** | **0.0106*** | 0.8600 | 0.0638 | 1.0000 | 0.0671 | / |
| **NNJD**  **n=10** | Na | 6.0000 | 3.0000 | 3.0000 | 6.0000 | 7.0000 | 2.0000 | 5.0000 | 6.0000 | 4.7500 |
|  | Ne | 3.6364 | 2.6316 | 2.8986 | 4.4444 | 5.0000 | 1.1050 | 3.4483 | 5.1282 | 3.5365 |
|  | Ho | 0.8000 | 0.0000 | 0.1000 | 0.5000 | 1.0000 | 0.1000 | 0.8000 | 0.4000 | 0.4625 |
|  | H_E_ | 0.7632 | 0.6526 | 0.6895 | 0.8158 | 0.8421 | 0.1000 | 0.7474 | 0.8474 | 0.6822 |
|  | P-HW | 0.9202 | **0.0001**** | **0.0003**** | **0.0010**** | 0.7545 | - | 0.2521 | **0.0114*** | / |
| **NNQC**  **n=12** | Na | 6.0000 | 3.0000 | 4.0000 | 4.0000 | 3.0000 | 4.0000 | 7.0000 | 6.0000 | 4.6250 |
|  | Ne | 3.4286 | 1.9459 | 2.5043 | 2.7961 | 2.1654 | 1.4187 | 4.8000 | 4.0563 | 2.8894 |
|  | Ho | 0.7500 | 0.0000 | 0.0833 | 0.6667 | 1.0000 | 0.3333 | 0.8333 | 0.5000 | 0.5208 |
|  | H_E_ | 0.7391 | 0.5072 | 0.6268 | 0.6703 | 0.5616 | 0.3080 | 0.8261 | 0.7862 | 0.6282 |
|  | P-HW | 1.0000 | **0.0001**** | **0.0000**** | 0.1284 | **0.0019**** | 1.0000 | 0.2233 | **0.0119*** | / |
| **HSJD**  **n=12** | Na | 7.0000 | 4.0000 | 8.0000 | 7.0000 | 5.0000 | 5.0000 | 4.0000 | 5.0000 | 5.6250 |
|  | Ne | 5.5385 | 3.2727 | 6.1277 | 5.7600 | 4.5714 | 2.5714 | 2.7170 | 3.3882 | 4.2434 |
|  | Ho | 0.9167 | 0.0000 | 0.2500 | 0.9167 | 0.5833 | 0.5000 | 0.5833 | 0.5833 | 0.5417 |
|  | H_E_ | 0.8551 | 0.7246 | 0.8732 | 0.8623 | 0.8152 | 0.6377 | 0.6594 | 0.7355 | 0.7704 |
|  | P-HW | 0.3163 | 0.0000****** | 0.0000****** | 0.8629 | 0.0876 | 0.0436 | 0.6308 | 0.459 | / |
| **HSFQ**  **n=12** | Na | 5.0000 | 2.0000 | 5.0000 | 5.0000 | 8.0000 | 2.0000 | 4.0000 | 5.0000 | 4.5000 |
|  | Ne | 4.1739 | 1.6000 | 3.7403 | 4.2353 | 5.7600 | 1.3846 | 2.0571 | 4.1739 | 3.3906 |
|  | Ho | 0.7500 | 0.0000 | 0.0833 | 0.5833 | 1.0000 | 0.0000 | 0.0000 | 0.5833 | 0.3750 |
|  | H_E_ | 0.7935 | 0.3913 | 0.7645 | 0.7971 | 0.8623 | 0.2899 | 0.5362 | 0.7935 | 0.6535 |
|  | P-HW | 0.0273***** | 0.0016****** | 0.0000****** | 0.2569 | 0.5387 | 0.0062****** | 0.0000****** | 0.0263 | / |
| **ZZJD**  **n=12** | Na | **5.0000** | **3.0000** | **7.0000** | **5.0000** | **7.0000** | **8.0000** | **8.0000** | **7.0000** | **6.2500** |
|  | Ne | 3.7403 | 1.8344 | 5.5385 | 3.2727 | 4.5714 | 4.2353 | 5.6471 | 5.8776 | 4.3396 |
|  | Ho | 0.5000 | 0.2500 | 0.5000 | 0.5000 | 0.8333 | 1.0000 | 0.8333 | 0.6667 | 0.6354 |
|  | H_E_ | 0.7645 | 0.4746 | 0.8551 | 0.7246 | 0.8152 | 0.7971 | 0.8587 | 0.8659 | 0.7695 |
|  | P-HW | 0.0772 | 0.0167 | 0.0081****** | 0.095 | 0.0535 | 0.5282 | 0.516 | 0.0034****** | / |
| **CXJD**  **n=12** | Na | **5.0000** | **3.0000** | **9.0000** | **8.0000** | **6.0000** | **8.0000** | **5.0000** | **4.0000** | **6.0000** |
|  | Ne | 3.0316 | 2.6667 | 6.6977 | 4.6452 | 4.9655 | 6.8571 | 3.7403 | 2.5043 | 4.3885 |
|  | Ho | 0.9167 | 0.0000 | 0.2500 | 0.4167 | 0.6667 | 0.5833 | 0.3333 | 0.5000 | 0.4583 |
|  | H_E_ | 0.6993 | 0.6522 | 0.8877 | 0.8188 | 0.8333 | 0.8913 | 0.7645 | 0.6268 | 0.7717 |
|  | P-HW | 0.8590 | 0.0000****** | 0.0000****** | 0.0000****** | 0.2025 | 0.0001****** | 0.0035****** | 0.4036 | / |
| **SQJD**  **n=12** | Na | 8.0000 | 3.0000 | 7.0000 | 7.0000 | 5.0000 | 6.0000 | 5.0000 | 5.0000 | 5.7500 |
|  | Ne | 3.7403 | 2.8800 | 4.3636 | 5.7600 | 3.2727 | 4.9388 | 3.1304 | 4.0563 | 4.0178 |
|  | Ho | 0.9167 | 0.0000 | 0.1667 | 0.8333 | 0.8333 | 0.8182 | 0.6667 | 0.6667 | 0.6127 |
|  | H_E_ | 0.7645 | 0.6812 | 0.8043 | 0.8623 | 0.7246 | 0.8355 | 0.7101 | 0.7862 | 0.7711 |
|  | P-HW | 0.7942 | 0.0000****** | 0.0000****** | 0.2594 | 1.0000 | 0.0848 | 0.9585 | 0.5573 | / |
| **CSJD**  **n=12** | Na | 9.0000 | 4.0000 | 6.0000 | 6.0000 | 3.0000 | 6.0000 | 6.0000 | 4.0000 | 5.5000 |
|  | Ne | 7.2000 | 3.7895 | 3.8400 | 3.2727 | 1.7669 | 4.5714 | 4.0563 | 2.2857 | 3.8478 |
|  | Ho | 0.3333 | 0.0000 | 0.1667 | 0.5833 | 0.0833 | 0.7500 | 0.5833 | 0.9167 | 0.4271 |
|  | H_E_ | 0.8986 | 0.7681 | 0.7717 | 0.7246 | 0.4529 | 0.8152 | 0.7862 | 0.5870 | 0.7255 |
|  | P-HW | 0.0000****** | 0.0000****** | 0.0000****** | 0.0890 | 0.0016****** | 0.0589 | 0.1136 | 0.0281***** | / |
| **WZJD**  **n=12** | Na | 7.0000 | 5.0000 | 7.0000 | 4.0000 | 5.0000 | 7.0000 | 4.0000 | 5.0000 | 5.5000 |
|  | Ne | 3.7895 | 3.9452 | 4.5000 | 2.7961 | 3.4699 | 4.0563 | 3.3882 | 3.7895 | 3.7168 |
|  | Ho | 0.5000 | 0.0833 | 0.8333 | 0.5000 | 1.0000 | 1.0000 | 0.5000 | 0.6667 | 0.6354 |
|  | H_E_ | 0.7681 | 0.7790 | 0.8116 | 0.6703 | 0.7428 | 0.7862 | 0.7355 | 0.7681 | 0.7577 |
|  | P-HW | 0.0070****** | 0.0000****** | 0.6565 | 0.0214***** | 0.0754 | 0.0244***** | 0.0393***** | 0.0817 | / |
| **CZJD**  **n=12** | Na | 5.0000 | 2.0000 | 8.0000 | 6.0000 | 6.0000 | 5.0000 | 6.0000 | 5.0000 | 5.3750 |
|  | Ne | 3.2727 | 1.9862 | 4.0563 | 3.0638 | 2.8235 | 3.0316 | 4.9655 | 2.5714 | 3.2214 |
|  | Ho | 0.5833 | 0.0833 | 0.3333 | 0.6667 | 0.6667 | 1.0000 | 0.7500 | 0.7500 | 0.6042 |
|  | H_E_ | 0.7246 | 0.5181 | 0.7862 | 0.7029 | 0.6739 | 0.6993 | 0.8333 | 0.6377 | 0.6970 |
|  | P-HW | 0.351 | 0.0044****** | 0.0001****** | 0.0173****** | 0.1139 | 0.0003****** | 0.0114***** | 0.4472 | / |
| **HZQC**  **n=12** | Na | 5.0000 | 3.0000 | 6.0000 | 7.0000 | 8.0000 | 3.0000 | 4.0000 | 7.0000 | 5.3750 |
|  | Ne | 3.0316 | 2.3226 | 2.8471 | 5.5385 | 3.8400 | 1.5238 | 2.3802 | 4.0000 | 3.1855 |
|  | Ho | 0.5833 | 0.0000 | 0.1818 | 0.7500 | 0.5833 | 0.0833 | 0.4167 | 0.7500 | 0.4186 |
|  | H_E_ | 0.6993 | 0.5942 | 0.6797 | 0.8551 | 0.7717 | 0.3587 | 0.6051 | 0.7826 | 0.6683 |
|  | P-HW | 0.2901 | 0.0000****** | 0.0009****** | 0.0220***** | 0.0038****** | 0.0062****** | 0.0287***** | 0.3175 | / |
| **HDJD**  **n=9** | Na | 5.0000 | 3.0000 | 4.0000 | 6.0000 | 2.0000 | 2.0000 | 6.0000 | 4.0000 | 4.0000 |
|  | Ne | 3.6818 | 2.4545 | 3.0000 | 4.2632 | 1.3846 | 1.1172 | 2.7931 | 2.7778 | 2.6840 |
|  | Ho | 0.4444 | 0.0000 | 0.0000 | 0.6667 | 0.3333 | 0.1111 | 0.5556 | 0.4000 | 0.3139 |
|  | H_E_ | 0.7712 | 0.6275 | 0.7059 | 0.8105 | 0.2941 | 0.1111 | 0.6797 | 0.7111 | 0.5889 |
|  | P-HW | 0.0191***** | 0.0002****** | 0.0000****** | 0.0318***** | 1.0000 | - | 0.0693 | 0.1429 | / |
| **HLJD**  **n=12** | Na | 4.0000 | 2.0000 | 3.0000 | 5.0000 | 1.0000 | 3.0000 | 4.0000 | 5.0000 | 3.3750 |
|  | Ne | 2.9691 | 1.9459 | 2.0000 | 2.2500 | 1.0000 | 1.1852 | 3.1648 | 4.1739 | 2.3361 |
|  | Ho | 0.5833 | 0.0000 | 0.0000 | 0.5833 | 0.0000 | 0.1667 | 0.6667 | 0.5000 | 0.3125 |
|  | H_E_ | 0.6920 | 0.5072 | 0.5217 | 0.5797 | 0.0000 | 0.1630 | 0.7138 | 0.7935 | 0.4964 |
|  | P-HW | 0.2303 | 0.0004****** | 0.0001****** | 0.3793 | 0.0000****** | 1.0000 | 0.0256***** | 0.0262***** | / |
| **SYJD**  **n=12** | Na | 4.0000 | 2.0000 | 5.0000 | 5.0000 | 6.0000 | 5.0000 | 5.0000 | 5.0000 | 4.6250 |
|  | Ne | 2.8515 | 1.8000 | 3.3882 | 4.1143 | 2.3607 | 2.7692 | 4.5000 | 3.6923 | 3.1845 |
|  | Ho | 0.6667 | 0.0000 | 0.0833 | 0.7500 | 0.5000 | 1.0000 | 0.6667 | 0.6667 | 0.5417 |
|  | H_E_ | 0.6775 | 0.4638 | 0.7355 | 0.7899 | 0.6014 | 0.6667 | 0.8116 | 0.7609 | 0.6884 |
|  | P-HW | 0.3059 | 0.0007****** | 0.0000****** | 0.6221 | 0.1579 | 0.0007****** | 0.0020****** | 0.5667 | / |
| **HBJD**  **n=12** | Na | 3.0000 | 6.0000 | 6.0000 | 2.0000 | 4.0000 | 6.0000 | 6.0000 | 2.0000 | 4.3750 |
|  | Ne | 1.4049 | 2.5263 | 4.6452 | 1.8000 | 2.9691 | 3.8919 | 2.1333 | 1.4922 | 2.6079 |
|  | Ho | 0.3333 | 0.2500 | 0.5833 | 0.0000 | 1.0000 | 1.0000 | 0.3333 | 0.4167 | 0.4896 |
|  | H_E_ | 0.3007 | 0.6304 | 0.8188 | 0.4638 | 0.6920 | 0.7754 | 0.5543 | 0.3442 | 0.5725 |
|  | P-HW | 1.0000 | 0.0012****** | 0.0864 | 0.0007****** | 0.0068****** | 0.1195 | 0.0177***** | 1.0000 | / |
| **HNJC**  **n=12** | Na | 5.0000 | 2.0000 | 4.0000 | 6.0000 | 6.0000 | 2.0000 | 4.0000 | 6.0000 | 4.3750 |
|  | Ne | 4.1143 | 1.3846 | 1.8824 | 4.4308 | 2.6422 | 1.1803 | 2.3040 | 4.1739 | 2.7641 |
|  | Ho | 0.9167 | 0.0000 | 0.0833 | 0.5833 | 0.5833 | 0.0000 | 0.3333 | 0.5000 | 0.3750 |
|  | H_E_ | 0.7899 | 0.2899 | 0.4891 | 0.8080 | 0.6486 | 0.1594 | 0.5906 | 0.7935 | 0.5711 |
|  | P-HW | 0.3859 | 0.0000****** | 0.0003****** | 0.0035****** | 0.2300 | 0.0435***** | 0.0621 | 0.0199****** | / |
| **JXQC**  **n=12** | Na | 5.0000 | 2.0000 | 4.0000 | 5.0000 | 5.0000 | 1.0000 | 6.0000 | 5.0000 | 4.1250 |
|  | Ne | 3.3488 | 1.9459 | 2.0571 | 4.0563 | 1.8947 | 1.0000 | 4.5000 | 2.6916 | 2.6868 |
|  | Ho | 0.5000 | 0.0000 | 0.0000 | 0.9167 | 0.2500 | 0.0000 | 0.7500 | 0.4167 | 0.3542 |
|  | H_E_ | 0.7319 | 0.5072 | 0.5362 | 0.7862 | 0.4928 | 0.0000 | 0.8116 | 0.6558 | 0.5652 |
|  | P-HW | 0.0964 | 0.0004****** | 0.0000****** | 0.1440 | 0.0486***** | - | 0.0780 | 0.0686 | / |
| **SQJDMZ**  **n=12** | Na | 1.0000 | 7.0000 | 1.0000 | 4.0000 | 2.0000 | 2.0000 | 6.0000 | 4.0000 | 3.3750 |
|  | Ne | 1.0000 | 4.8000 | 1.0000 | 2.1985 | 1.1803 | 1.3846 | 3.1304 | 3.3882 | 2.2603 |
|  | Ho | 0.0000 | 0.4167 | 0.0000 | 0.3333 | 0.0000 | 0.0000 | 0.8333 | 0.7500 | 0.2917 |
|  | H_E_ | 0.0000 | 0.8261 | 0.0000 | 0.5688 | 0.1594 | 0.2899 | 0.7101 | 0.7355 | 0.4112 |
|  | P-HW | - | 0.0004****** | - | 0.0495***** | 0.0435***** | 0.0062****** | 1.0000 | 0.3989 | / |
| **SQNGMZ**  **n=12** | Na | 2.0000 | 4.0000 | 1.0000 | 4.0000 | 1.0000 | 2.0000 | 6.0000 | 7.0000 | 3.3750 |
|  | Ne | 1.3846 | 3.4699 | 1.0000 | 2.1654 | 1.0000 | 1.1803 | 1.7349 | 3.3882 | 1.9154 |
|  | Ho | 0.0000 | 0.2500 | 0.0000 | 0.5833 | 0.0000 | 0.0000 | 0.5000 | 1.0000 | 0.2917 |
|  | H_E_ | 0.2899 | 0.7428 | 0.0000 | 0.5616 | 0.0000 | 0.1594 | 0.4420 | 0.7355 | 0.3664 |
|  | P-HW | 0.0062****** | 0.0004****** | - | 1.0000 | - | 0.0435***** | 1.0000 | 0.0095****** | / |
| **HNSGMZ**  **n=10** | Na | 1.0000 | 4.0000 | 1.0000 | 5.0000 | 1.0000 | 3.0000 | 7.0000 | 5.0000 | 3.3750 |
|  | Ne | 1.0000 | 3.0769 | 1.0000 | 2.1277 | 1.0000 | 1.6529 | 3.8462 | 3.8462 | 2.1937 |
|  | Ho | 0.0000 | 0.1000 | 0.0000 | 0.5000 | 0.0000 | 0.1000 | 0.7000 | 0.6000 | 0.2500 |
|  | H_E_ | 0.0000 | 0.7105 | 0.0000 | 0.5579 | 0.0000 | 0.4158 | 0.7789 | 0.7789 | 0.4053 |
|  | P-HW | - | 0.0001****** | - | 0.05858 | - | 0.0093****** | 0.4379 | 0.3948 | / |
| **HNJDFQ**  **n=12** | Na | 3.0000 | 2.0000 | 4.0000 | 11.0000 | 9.0000 | 6.0000 | 11.0000 | 1.0000 | 5.8750 |
|  | Ne | 1.4118 | 1.1803 | 1.5568 | 6.5455 | 7.2000 | 3.8919 | 8.4706 | 1.0000 | 3.9071 |
|  | Ho | 0.1667 | 0.0000 | 0.0833 | 0.8333 | 0.2500 | 1.0000 | 0.9167 | 0.0000 | 0.4062 |
|  | H_E_ | 0.3043 | 0.1594 | 0.3732 | 0.8841 | 0.8986 | 0.7754 | 0.9203 | 0.0000 | 0.5394 |
|  | P-HW | 0.089 | 0.0435***** | 0.0021****** | 0.5346 | 0.0000****** | 0.1170 | 0.5576 | - | / |
| **NMB**  **n=6** | Na | 1.0000 | 2.0000 | 0.0000 | 9.0000 | 5.0000 | 2.0000 | 3.0000 | 3.0000 | 3.5714 |
|  | Ne | 1.0000 | 2.0000 | 0.0000 | 6.0000 | 4.5455 | 1.3846 | 2.3226 | 2.5714 | 2.8320 |
|  | Ho | 0.0000 | 0.0000 | 0.0000 | 0.6667 | 0.2000 | 0.0000 | 1.0000 | 0.0000 | 0.2667 |
|  | H_E_ | 0.0000 | 0.5455 | 0.0000 | 0.9091 | 0.8667 | 0.3030 | 0.6212 | 0.6667 | 0.5589 |
|  | P-HW | - | - | - | 0.0247***** | 0.0031****** | 0.0909 | 0.0909 | 0.0043****** | / |
| **CB**  **n=6** | Na | 3.0000 | 2.0000 | 2.0000 | 6.0000 | 6.0000 | 2.0000 | 1.0000 | 3.0000 | 3.1250 |
|  | Ne | 1.6744 | 1.1803 | 1.3846 | 3.7895 | 6.0000 | 1.3846 | 1.0000 | 1.6744 | 2.2610 |
|  | Ho | 0.1667 | 0.1667 | 0.0000 | 0.5000 | 0.0000 | 0.0000 | 0.0000 | 0.1667 | 0.1250 |
|  | H_E_ | 0.4394 | 0.1667 | 0.3030 | 0.8030 | 0.9091 | 0.3030 | 0.0000 | 0.4394 | 0.4205 |
|  | P-HW | 0.0909 | 0.0216***** | 0.0909 | 0.0760 | 0.0000****** | 0.0909 | - | 0.0909 | / |

N_a_, number of alleles; N_e_, effective number of alleles; *H_O_*, observed heterozygosity; *H_E_*, expected heterozygosity; PIC, Polymorphic information content; P-HW, value from the exact test for Hardy–Weinberg equilibrium; *denotes a significant deviation from Hardy–Weinberg equilibrium (P﹤0.01), after sequential Bonferroni’s correction of the significance threshold.

**Supplementary Table S4. Pairwise F_ST_ (below the diagonal) and P value (above the diagonal) of five *Liriomyza* species based on eight microsatellite locis**

|  | **HLJD** | **NNQC** | **SYJD** | **HBJD** | **DGQC** | **CZJD** | **SQJD** | **CXJD** | **CSJD** | **HSJD** | **HNJDFQ** | **ZZJD** | **BLJD** | **WZJD** | **HSFQ** | **NNJD** | **HNSGM** | **HDJD** | **HZQC** | **JXQC** | **HNJC** | **SQJDM** | **SQNGM** | **NMB** | **CB** |
| --- | --- | --- | --- | --- | --- | --- | --- | --- | --- | --- | --- | --- | --- | --- | --- | --- | --- | --- | --- | --- | --- | --- | --- | --- | --- |
| **HLJD** |  | 0.00000 | 0.00000 | 0.00000 | 0.00000 | 0.00000 | 0.00000 | 0.00000 | 0.00000 | 0.00000 | 0.00000 | 0.00000 | 0.00000 | 0.00000 | 0.00000 | 0.00000 | 0.00000 | 0.00901 | 0.00000 | 0.00000 | 0.00000 | 0.00000 | 0.00000 | 0.00000 | 0.00000 |
| **NNQC** | 0.11992 |  | 0.00000 | 0.00000 | 0.00000 | 0.00000 | 0.00000 | 0.00000 | 0.00000 | 0.00000 | 0.00000 | 0.00000 | 0.00000 | 0.00000 | 0.00000 | **0.05405** | 0.00000 | 0.00000 | 0.00000 | 0.00901 | 0.00000 | 0.00000 | 0.00000 | 0.00000 | 0.00000 |
| **SYJD** | 0.20762 | 0.20258 |  | 0.00000 | 0.00000 | 0.02703 | 0.00000 | 0.00000 | 0.00000 | 0.00000 | 0.00000 | 0.00000 | 0.00000 | 0.00000 | 0.00000 | 0.00000 | 0.00000 | 0.00000 | 0.00000 | 0.00000 | 0.00000 | 0.00000 | 0.00000 | 0.00000 | 0.00000 |
| **HBJD** | **0.27185** | **0.26370** | 0.21991 |  | 0.00000 | 0.00000 | 0.00000 | 0.00000 | 0.00000 | 0.00000 | 0.00000 | 0.00000 | 0.00000 | 0.00000 | 0.00000 | 0.00000 | 0.00000 | 0.00000 | 0.00000 | 0.00000 | 0.00000 | 0.00000 | 0.00000 | 0.00000 | 0.00000 |
| **DGQC** | 0.21427 | 0.18090 | 0.07934 | 0.17375 |  | 0.00000 | 0.00000 | 0.00000 | 0.00000 | 0.00000 | 0.00000 | 0.00000 | **0.06306** | 0.00000 | 0.00000 | 0.00000 | 0.00000 | 0.00000 | 0.00000 | 0.00000 | 0.00000 | 0.00000 | 0.00000 | 0.00000 | 0.00000 |
| **CZJD** | 0.15554 | 0.14066 | 0.04745 | 0.16528 | 0.07216 |  | 0.00000 | 0.00000 | 0.00000 | 0.00000 | 0.00000 | 0.00901 | 0.00000 | 0.00000 | 0.00000 | 0.00000 | 0.00000 | 0.00000 | 0.00000 | 0.00000 | 0.00000 | 0.00000 | 0.00000 | 0.00000 | 0.00000 |
| **SQJD** | 0.22490 | 0.18985 | 0.15152 | 0.21972 | 0.18209 | 0.13319 |  | **0.09910** | 0.00000 | 0.00000 | 0.00000 | 0.00000 | 0.00000 | 0.00000 | 0.00000 | 0.00000 | 0.00000 | 0.00000 | 0.00000 | 0.00000 | 0.00000 | 0.00000 | 0.00000 | 0.00000 | 0.00000 |
| **CXJD** | 0.20389 | 0.16484 | 0.12300 | 0.18176 | 0.15494 | 0.08491 | 0.03738 |  | 0.00000 | 0.03604 | 0.00000 | 0.00000 | 0.00000 | 0.00000 | 0.00000 | 0.00000 | 0.00000 | 0.00000 | 0.00000 | 0.00000 | 0.00000 | 0.00000 | 0.00000 | 0.00000 | 0.00000 |
| **CSJD** | 0.18539 | 0.17727 | 0.12614 | 0.19456 | 0.16392 | 0.09234 | 0.13388 | 0.09669 |  | 0.00000 | 0.00000 | 0.00000 | 0.00000 | 0.00000 | 0.00000 | 0.00000 | 0.00000 | 0.00000 | 0.00000 | 0.00000 | 0.00000 | 0.00000 | 0.00000 | 0.00000 | 0.00000 |
| **HSJD** | 0.21395 | 0.16866 | 0.10300 | 0.19743 | 0.14663 | 0.07853 | 0.05174 | 0.04719 | 0.06748 |  | 0.00000 | 0.00000 | 0.00000 | 0.00000 | 0.00000 | 0.00000 | 0.00000 | 0.00000 | 0.00000 | 0.00000 | 0.00000 | 0.00000 | 0.00000 | 0.00000 | 0.00000 |
| **HNJDFQ** | **0.45139** | **0.35443** | **0.33563** | **0.39837** | **0.35068** | **0.30486** | **0.31443** | **0.31313** | **0.33718** | **0.29869** |  | 0.00000 | 0.00000 | 0.00000 | 0.00000 | 0.00000 | 0.00000 | 0.00000 | 0.00000 | 0.00000 | 0.00000 | 0.00000 | 0.00000 | 0.00000 | 0.00000 |
| **ZZJD** | 0.21557 | 0.15680 | 0.06851 | 0.18119 | 0.05287 | 0.04419 | 0.10907 | 0.10981 | 0.11406 | 0.08749 | **0.28876** |  | **0.27027** | 0.00000 | 0.00000 | 0.00000 | 0.00000 | 0.00000 | 0.00000 | 0.00000 | 0.00000 | 0.00000 | 0.00000 | 0.00000 | 0.00000 |
| **BLJD** | 0.22301 | 0.17660 | 0.08725 | 0.15378 | 0.02928 | 0.08302 | 0.15243 | 0.14094 | 0.12806 | 0.11667 | **0.32696** | **0.01444** |  | 0.00000 | 0.00000 | 0.00000 | 0.00000 | 0.00000 | 0.00000 | 0.00000 | 0.00000 | 0.00000 | 0.00000 | 0.00000 | 0.00000 |
| **WZJD** | 0.18402 | 0.14189 | 0.11529 | 0.10832 | 0.10765 | 0.05213 | 0.10643 | 0.08520 | 0.09641 | 0.09841 | **0.29995** | 0.05286 | 0.05167 |  | 0.00000 | 0.00000 | 0.00000 | 0.00000 | 0.00000 | 0.00000 | 0.00000 | 0.00000 | 0.00000 | 0.00000 | 0.00000 |
| **HSFQ** | 0.15490 | 0.14355 | 0.18615 | **0.30116** | 0.21813 | 0.15144 | 0.14632 | 0.16165 | 0.17513 | 0.12234 | **0.34652** | 0.13947 | 0.18703 | 0.18428 |  | 0.00000 | 0.00000 | 0.00901 | 0.00000 | 0.00000 | 0.00000 | 0.00000 | 0.00000 | 0.00000 | 0.00000 |
| **NNJD** | 0.13575 | 0.04457 | 0.19660 | 0.19826 | 0.16183 | 0.15131 | 0.16363 | 0.13677 | 0.17130 | 0.14364 | **0.33792** | 0.13414 | 0.15064 | 0.12006 | 0.11476 |  | 0.00000 | 0.00000 | 0.00901 | 0.00000 | 0.00000 | 0.00000 | 0.00000 | 0.00000 | 0.00000 |
| **HNSGMZ** | **0.40106** | **0.35204** | **0.37763** | **0.47556** | **0.40144** | **0.37266** | **0.38582** | **0.34084** | **0.31986** | **0.34934** | **0.51793** | **0.35598** | **0.36723** | **0.35335** | **0.36197** | **0.37139** |  | 0.00000 | 0.00000 | 0.00000 | 0.00000 | **0.97297** | 0.00901 | 0.00000 | 0.00000 |
| **HDJD** | 0.10383 | 0.13255 | 0.16377 | **0.30854** | 0.18821 | 0.12603 | 0.23563 | 0.20705 | 0.17328 | 0.19516 | **0.38919** | 0.15746 | 0.17407 | 0.16303 | 0.12034 | 0.13292 | **0.25708** |  | **0.11712** | **0.51351** | 0.00000 | 0.00000 | 0.00000 | 0.00000 | 0.00000 |
| **HZQC** | 0.10302 | 0.08905 | 0.15858 | 0.21739 | 0.09083 | 0.11867 | 0.18277 | 0.15811 | 0.17201 | 0.17227 | **0.37115** | 0.09995 | 0.09844 | 0.10444 | 0.13453 | 0.05984 | **0.34181** | 0.05849 |  | 0.00901 | **0.48649** | 0.00000 | 0.00000 | 0.00000 | 0.00000 |
| **JXQC** | 0.10701 | 0.12462 | 0.18131 | **0.33640** | 0.20088 | 0.13344 | 0.23338 | 0.21138 | 0.20949 | 0.20955 | **0.39862** | 0.17535 | 0.19685 | 0.18568 | 0.11947 | 0.13825 | **0.36183** | **0.01923** | 0.06735 |  | 0.00000 | 0.00000 | 0.00000 | 0.00000 | 0.00000 |
| **HNJC** | 0.10336 | 0.10259 | 0.22944 | **0.26546** | 0.16915 | 0.16426 | 0.23093 | 0.19971 | 0.22963 | 0.22788 | **0.41916** | 0.18868 | 0.18077 | 0.14696 | 0.19796 | 0.08456 | **0.37840** | 0.09181 | **0.01581** | 0.07781 |  | 0.00000 | 0.00000 | 0.00000 | 0.00000 |
| **SQJDMZ** | **0.40677** | **0.35883** | **0.39097** | **0.48041** | **0.41111** | **0.38635** | **0.39530** | **0.35657** | **0.33944** | **0.36063** | **0.51724** | **0.36574** | **0.37729** | **0.36838** | **0.36609** | **0.37386** | -0.02133 | **0.26322** | **0.34786** | **0.36698** | **0.38093** |  | **0.07207** | 0.00000 | 0.00000 |
| **SQNGMZ** | **0.42281** | **0.37883** | **0.41284** | **0.50284** | **0.42886** | **0.41385** | **0.42376** | **0.38861** | **0.36227** | **0.38734** | **0.53808** | **0.38883** | **0.39528** | **0.39090** | **0.38932** | **0.39009** | 0.08675 | **0.29717** | **0.36450** | **0.38865** | **0.40166** | 0.04547 |  | 0.00000 | 0.00901 |
| **NMB** | **0.28086** | 0.19789 | **0.26106** | **0.34348** | **0.26026** | 0.23237 | 0.21548 | 0.21874 | 0.24664 | 0.21645 | **0.31395** | 0.23495 | **0.25329** | 0.21874 | 0.18575 | 0.16439 | **0.41357** | 0.17765 | 0.17417 | 0.17557 | 0.19235 | **0.40783** | **0.43991** |  | 0.00000 |
| **CB** | **0.46794** | **0.38809** | **0.39214** | **0.47011** | **0.40488** | **0.38574** | **0.37057** | **0.36359** | **0.39087** | **0.36274** | **0.47883** | **0.34788** | **0.36260** | **0.35822** | **0.37148** | **0.34761** | **0.52699** | **0.39837** | **0.36279** | **0.38171** | **0.38963** | **0.51202** | **0.54213** | **0.37391** |  |

**Supplementary Table S5. Primer sequences of COI and EF-1a**

| **Gene** | **Sequence** | **Source** |
| --- | --- | --- |
| **COI** | forward：5’-CAACATTTATTTTGATTTTTT GG-3’ | Simon *et al*，1994 |
|  | reword：5’-TCCAATGCACTAATCTGCCATATT A-3’ |  |
| **EF-1a** | forward：5’-CGTGGTATTACCATCGATATTGC-3’ | Designed by our team |
|  | forward：5’-TGTGGACTGGGCCTTTGAGTCA-3’ |  |

**Supplementary Table S6.** [**Information**](app:ds:information) **of eight polymorphic microsatellite locis**

| **Locus** | **Primer sequences （5’-3’）** | **Fluorophore** | **Ta（℃）** |
| --- | --- | --- | --- |
| **SSR-6** | F: CGAGGACCAAGTTATAGTGGAGG | FAM | 53 |
|  | R: TGCAACATACACAAAGCAGCAAT |  |  |
| **SSR-24** | F: TCCACTACTACTTGTACCACTGC | HEX | 53 |
|  | R: TGTGGCAATTCAGGAACATAGGA |  |  |
| **SSR-26** | F: CCTACTCCTGCACCAATTCGTAT | ROX | 58 |
|  | R: GTCAACTTGACTTGTCAGTTGCA |  |  |
| **SSR-35** | F: GAGTGCGATTCCAATAGCTAGGA | TAMRA | 58 |
|  | R: GGTTCATTCCGTTGTTATCGTCG |  |  |
| **SSR-39** | F: GCCTGGGTTATTGATGAGGTCTT | ROX | 58 |
|  | R: GTTACATCAACTTCTACTGCCGC |  |  |
| **SSR-40** | F: CACGCAAGGCAATGATGTTCTTA | TAMRA | 58 |
|  | R: CCAACAAGCCGTTAGTCCAAGTA |  |  |
| **JY46** | F:TATACCTCTGTGGAATGGG | HEX | 51 |
|  | R:AGTAAGCATACCTCAGCAT |  |  |
| **JY70B** | F:AATCAAGATGAGGAAATAAG | HEX | 51 |
|  | R:TTTCAAATCGCCTACAA |  |  |
